# Fertilizer Addition Modifies Utilization of Different P Sources in Upland Rice on Strongly P-fixing Andosols

**DOI:** 10.1007/s42729-024-01774-1

**Published:** 2024-05-07

**Authors:** Eva Mundschenk, Rainer Remus, Jürgen Augustin, Matthias Wissuwa, Christiana Staudinger, Eva Oburger, Eckhard George, Maire Holz

**Affiliations:** 1 Group of Isotope Biogeochemistry and Gas Fluxes, Leibniz Centre for Agricultural Landscape Research (ZALF) E.V., Müncheberg, Germany; 2 Japan International Research Center for Agricultural Sciences (JIRCAS), 1-1 Ohwashi, Tsukuba, Ibaraki 16 305-8686 Japan; 3 Department of Forest and Soil Science, Institute of Soil Research, University of Natural Resources and Life Sciences, 3430 Tulln an Der Donau, Austria; 4 Department of Crop Sciences, Institute of Soil Research, University of Natural Resources and Life Sciences, 3430 Tulln an Der Donau, Austria; 5 Department of Crop and Animal Sciences, Plant Nutrition, Humboldt University, Unter Den Linden 6, 10099 Berlin, Germany; 6 Present Address: PhenoRob Cluster & Institute of Crop Science and Resource Conservation, Bonn University, Bonn, Germany

**Keywords:** Phosphorus acquisition efficiency, Andosol, Upland rice, ^33^P labeling, Fertilizer-P, Native soil-P

## Abstract

**Aims:**

High Phosphorus (P) efficiencies such as internal P utilization efficiency (PUE) and P acquisition efficiency (PAE) are crucial for upland rice production, particularly on highly P-fixing soils like Andosols. While the effect of root traits associated with high PAE in upland rice has been studied intensively, less attention has been given to the origin of P (native soil-P versus fertilizer-P) taken up by plants when evaluating differences in P efficiency. Here we aim to evaluate the efficiency of different upland rice genotypes to acquire native soil-P and fertilizer-P.

**Methods:**

Four upland rice genotypes with varying PAE were grown in an Andosol at low- and high-P fertilization level and harvested 9 and 34 days after emergence. Fertilizer-P was labeled with ^33^P to distinguish between the efficiency to acquire P originating from native soil and fertilizer by measuring plant P uptake.

**Results:**

Increased fertilizer supply enhanced native soil-P uptake. Under low-P conditions the genotype DJ123 showed a superior PAE and an increased acquisition of native soil-P while AB199 was identified to have a superior internal PUE under P deficient conditions. Differences between genotypes in overall PAE under high-P conditions were not significant but the distinction of P sources showed that genotype DJ123 acquired significantly more native soil-P per unit root than all other genotypes.

**Conclusions:**

Our results indicate that variations in PAE among genotypes are associated with their ability to access native soil-P. DJ123 emerged as the most adept genotype in acquiring sparingly soluble native soil-P and future studies should unravel the rhizosphere processes underlying increased PAE of native soil-P.

**Supplementary Information:**

The online version contains supplementary material available at 10.1007/s42729-024-01774-1.

## Introduction

Volcanic ash soils, known as Andosols, are widespread across the globe and are commonly characterized by a porous soil structure, high water-holding capacity and a high nutrient density (Nanzyo 2002). These qualities make them highly suitable for agriculture, supporting the livelihoods of many people worldwide (Shoji et al. 1993; Takahashi and Shoji 2002). Despite their high total nutrient content, Andosols often have low plant availability of phosphorus (P) (Otani and Ae 1996). This limitation is primarily due to the presence of active Al- and Fe- (hydr-)oxides and organo-Al- and Fe-complexes, which have a strong affinity for phosphate adsorption (Otani and Ae 1999; Balemi and Negisho 2012; Hashimoto et al. 2012).

The challenge of limited P availability becomes especially pronounced when economic constraints hinder the extensive application of P fertilizers, a circumstance frequently encountered in upland rice cultivation on soils with high P fixation (Hedley et al. 1994; Atakora et al. 2015). This situation is aggravated by the depletion of rock phosphate resources, further emphasizing the need for sustainable approaches in managing P in agriculture (Otani and Ae 1996; Simpson et al. 2011; Ulrich and Frossard 2014). Therefore, breeding for P-efficient genotypes is a common approach to address the challenges posed by high P fixing soils and low fertilizer accessibility (Rose and Wissuwa 2012). Plants employ two primary, non-exclusive strategies to augment P acquisition: P foraging and soil-bound P mining (Richardson et al. 2011; Chen et al. 2023). The P foraging strategy involves maximizing the exploration of soil volume by the root system to efficiently capture as much plant-available P as possible (Adesemoye and Kloepper 2009; Nestler and Wissuwa 2016) and is particularly crucial in soils where P diffusion is limited. In addition, plants exhibit the ability to mine P that is not readily available for uptake. This capacity is quantified as the P acquisition efficiency (PAE), expressed as the amount of P absorbed per unit of root size (Mori et al. 2016; Wissuwa et al. 2020). This metric provides valuable insights into a p lant's effectiveness in extracting P from less accessible soil-bound sources efficiency (Richardson et al. 2011; Kuppe et al. 2022; Chen et al. 2023), contributing to a comprehensive understanding of its nutrient acquisition.

It has furthermore been suggested to explore genotypic differences in internal P utilization efficiency (PUE) which has been defined as the biomass produced per unit P (Rose and Wissuwa 2012). The key determinants of elevated PUE involve the effective re-mobilization and re-translocation of P, predominantly from older tissues to newly developing ones, hereby optimizing nutrient utilization within the plant. Specifically, examining genotypic disparities in root PUE becomes pertinent, as the P remobilized from aging roots (or leaves) can potentially be allocated towards fueling the expansion of the root system (Aerts 1996; Veneklaas et al. 2012).

While the ability and the mechanisms of plant species to mobilize sparingly available P sources has been studied extensively (Wang et al. 2010b; Nishigaki et al. 2019), there is a notable lack of information regarding the efficiency of P acquisition from various P sources in the soil (soil vs. fertilizer-P). Hedley et al. (1994) evaluated the ability of upland rice genotypes to acquire native soil and fertilizer-P and found no genotypic differences between the cultivars studied. Shimamura et al. (2021) found genotypic differences in PAE between six rice varieties under moderate P deficiency but the authors observed no differences in the efficiency of those genotypes to acquire organic-P.

Furthermore, it is widely recognized that a minimum supply of P is essential for initial plant growth and serves as the foundation for the plant to access and acquire further P resources (Grant et al. 2001, 2005; Richardson et al. 2009). However, because breeding efforts for PAE primarily target low-input systems without fertilizer addition, there is a notable gap in the literature to our knowledge regarding the response of different genotypes varying in PAE to varying fertilization rates. The identification of genotypes that respond well to low fertilizer-P application while enhancing native soil-P acquisition aims to optimize productivity and resource efficiency in rice cultivation within low-input systems.

In order to address these uncertainties, we grew four different upland rice genotypes in an Andosol with a low and a sufficient P fertilizer supply. We investigated the P-efficient genotype DJ123, the P-inefficient genotype Nerica4 and two promising sister lines of both of them (AB199 and AB67) (Koide et al. 2013; Wissuwa et al. 2020; Ranaivo et al. 2022). The relative contribution of different P sources (native soil-P versus freshly applied P fertilizer) to total P uptake by the different rice lines was quantified using ^33^P labeled fertilizer. With the use of ^33^P, we can distinctly differentiate between the uptake of native soil-P and fertilizer-P. An early harvest date (9 days after emergence) was included in the experiment to observe and adjust for the initiation of P uptake beyond seed-P. The main harvest date was set to be 34 DAE, coinciding with the beginning of tillering. Native soil-P in this study refers to the indigenous and residual soil-P (non-radioactive P), i.e. all P forms that were in the soil before the start of the experiment, whereas “freshly” applied P refers to the fertilizer-P applied at the beginning of the experiment.

Our objectives encompassed three key aspects: firstly, to evaluate the PAE and PUE across various tested genotypes; secondly, to establish connections between the variations in PAE and the efficiencies in acquiring native soil-P versus fresh fertilizer-P; and lastly, to investigate whether the introduction of a high dose of P fertilizer negates the observed effects of PAE.

## Materials and Methods

### Soil

Two pot experiments were conducted with a P-deficient Andosol collected from an upland field site at the experimental station of JIRCAS located in Tsukuba, Japan (36°03′09.6"N 140°04′39.2"E). The soil texture of the Andosol was distributed as follows: clay: 11%, silt: 50% and sand: 39%. The soil had a total P concentration of 930 mg kg^−1^ and a plant available P (Bray 2) concentration of 7 mg P kg^−1^ (Table 1). Prior to the experiment the soil was air dried and sieved to < 1 mm in order to allow for a homogenous fertilizer distribution into the soil.

**Table 1:** Soil properties of the Andosol used for the two pot experiments

	pH	C_Total_	N_Total_	P_Total_	Bray-2 P	P_ox_	Al_ox_	Fe_ox_
	[H_2_O]	[g kg^−1^]	[g kg^−1^]	[mg kg^−1^]	[mg kg^−1^]	[mg kg^−1^]	[g kg^−1^]	[g kg^−1^]
Andosol	5.88	42.6	3.14	930	7	677.8	39.6	11.6
	± 0.14	± 0.68	± 0.084	± 6	± 0.54	± 27.8	± 0.375	± 0.118

The values are means ± standard deviation. pH analyzed according to DIN ISO 10390; C_Total_ analyzed according to DIN ISO 10694; N_Total_ analyzed according to DIN ISO 13878; P_Total_ according to König (2005); Bray-2 according to Bray and Kurtz (1945) P_ox_—oxalate-extractable P, Al_ox_—oxalate-extractable Al and Fe_ox_—oxalate-extractable Fe according to König (2005)

### Sorption Experiment

In order to test the fertilizer-P sorption of the Andosol used, a sorption experiment was performed. For this, 2 kBq ^33^P-labeled phosphoric acid (H_3_^33^PO_**4**_) (Hartmann Analytic GmbH, Brunswick, Germany) were mixed with a 24-fold concentrated low- (3.5 mg P kg^−1^) and high-P (70 mg P kg^−1^) solution (both single nutrient solutions contained disodium phosphate (Na_2_HPO_4_)). The substrates employed in the sorption comprised the Andosol, an acid-washed quartz sand, and a sandy soil from Müncheberg, Germany, used for comparative analysis (Suppl. Table S1). Then 500 mg of each substrate were separately mixed with 1200 µl spiked ^33^P-nutrient solution and shaken for 60 min on a horizontal shaker. The tubes were subsequently centrifuged at 25 830 g for 5 min at 4 °C. Of each tube, 600 µl were taken from the supernatant and mixed with 5 ml of UltimaGold-Scintillator (PerkinElmer, USA) and ^33^P was quantified using a liquid scintillation counter (LSC; TriCarb 2800 TR, PerkinElmer, Rodgau, Germany). Sorption of ^33^P to the soil was then calculated in relation to the sorption of ^33^P to the control.

### Rice Varieties

For the pot experiment, four different upland rice varieties (*Oryza sativa* L.) with contrasting PAE were chosen. Genotype DJ123 belonging to the *aus* subspecies of rice is known to have high PAE (Mori et al. 2016; Wissuwa et al. 2020) whereas the African rice variety Nerica4 was chosen for its low PAE (Koide et al. 2013; Mori et al. 2016). Breeding lines AB67 and AB199 are sister lines derived from a cross of contrasting parents DJ123 and Nerica4 and both showed high PAE in prior field experiments (Matthias Wissuwa, unpublished data).

### Pot Experiment and Growth Conditions

The experiment involved the aforementioned four upland rice genotypes, which were subjected to two fertilizer levels and harvested at two different dates. Nitrogen (N), P and potassium (K) in the nutrient solutions were supplied as ammonium nitrate (NH_4_NO_3_), disodium phosphate (Na_2_HPO_4_), and potassium sulfate (K_2_SO_4_), respectively. The fertilizer treatments consisted of a high-P (N:P:K, 100:70:100 mg kg-1) and a low-P treatment (N:P:K, 100:3.5:100 mg kg-1). The term high-P treatment, as used in this study, denotes a P fertilizer treatment that is not genuinely high but rather moderate. This designation is based on the analysis of moderate P concentrations in the plant tissues at the end of the experiment (Yoshida 1981; Hedley et al. 1994). To label the soil with ^33^P, phosphoric acid (H_3_^33^PO_4_) (Hartmann Analytic GmbH, Brunswick, Germany), was added to each nutrient solution. For the first harvest at 9 days after emergence (DAE), 355 g of soil and 1.75 MBq of ^33^P per pot were used, while for the second harvest at 34 DAE, 700 g of soil and 6.6 MBq of ^33^P per pot were supplied, considering the short half-life of ^33^P (t_1/2_ 25.34 days). The ^33^P-spiked nutrient solutions were homogenized into the soil using a handheld electric mixer. The choice of the first harvest date at 9 DAE was based on the expectation that P uptake from the soil would have surpassed seed-P translocation in importance at this point (Julia et al. 2018). After adding the fertilizer to the soil, the soil was dried over night at 65 °C and was filled into the pots on the next day and watered to maximum water holding capacity (max WHC: 70%, estimated according to DIN ISO 11274). Afterwards, one seed per pot was placed directly into the soil. The volumetric water content was kept at 28 – 31% during plant growth (40–45% of max WHC). Four replicates of each treatment combination were grown in a completely randomized design in a climate chamber. The temperature was 30 °C during the day and 24 °C during the night. The photoperiod was 14 h and the light intensity was 300 µmol m^−2^ s^−1^.

### Plant and Soil Analysis

At 9 and 34 DAE, shoots were cut and removed directly above the soil surface. After shoot harvest, the roots were taken out of the soil and loose roots in the remaining soil were picked with a tweezer. Collected roots were gently washed in deionized water and adhering soil was removed and defined as rhizosphere soil (RS). Soil with no visible roots was considered as bulk soil (BS). Root and shoot biomass were determined after oven drying at 65 °C for 72 h. Subsequently, all plant samples were ground to a fine powder using a high-speed ball mill (Retsch M 400, Haan, Germany). Shoot and root tissue P concentration was determined after pressure digestion in 2 ml of 64% HNO_3_ (König 2005) followed by the determination of P concentrations in a microplate reader (SpectraMax High, Molecular Devices, USA) at 711 nm wavelength using the modified molybdenum blue assay by Murphy and Riley (1962) as described by Tiessen and Moir (2008). Total soil-P (TP) was determined from the bulk and rhizosphere of 0.05 g soil after pressure digestion as described for plant analysis. Seed-P content was determined by digesting 5 dehulled seeds per genotype as described for plant and soil analysis and by calculating the 1000 kernel weight. We assumed that at the first harvest 70% and at the second harvest 85% of the seed-P was translocated into the seedling (M. Wissuwa, personal communication).

### ^33^P Determination in Plant and Soil Samples and Calculations

^33^P activity in all pressure digested plant and soil extracts was determined using liquid scintillation counting. Briefly, 1 ml of the extract was mixed with 10 ml of scintillation cocktail (Rotiszint eco high, Roth, Karlsruhe, Germany) and measured on a liquid scintillation counter (Tri-Carb® 2800TR, Perkin Elmer, Germany). To take the decay time between the setup of experiments and measurement into account the ^33^P signal was corrected for the ^33^P half-life (t_1/2_) of 25.34 days on the reference day of each radioactive source (Eq. 1) with λ representing the decay constant.

1 $${\mathrm{t}}_{\frac{1}{{2}}}=\frac{{\mathrm{ln}}\left(2\right)}{\lambda }$$

The specific ^33^P activity (S.A. in kBq ^33^P mg P^−1^) of a sample (shoot and root) was calculated according to IAEA (2001):

2 $$\mathrm{S.A.}\left[{{\mathrm{kBq}} \, {}^{33}{\mathrm{P}} \, {\mathrm{mg}}\mathrm{ P}}^{-1}\right]\mathrm{ } = \frac{{}^{33}{\mathrm{P}} \, \left[{\mathrm{kBq}}{ \, {\mathrm{tissue}}}^{-1}\right]}{{\mathrm{P}} \, \left[{\mathrm{mg\; P\; tissue}}^{-1}\right]}$$

Total P uptake per pot was calculated by subtracting the assumed quantity of seed-P translocated to root and shoot tissue (i.e. 70% at 9 DAE and 85% at 34 DAE) of the respective genotype from the total plant P content per pot as shown in Eq. 3:

3 $$\mathrm{Total \;P\; uptake}=\mathrm{Plant\; P \;content }\left[\mathrm{\mu g \;P }\;{{\mathrm{plant}}}^{-1}\right]-{\text{Seed-P}}\mathrm{\;content }\left[\mathrm{\mu g\; P }\;{{\mathrm{seed}}}^{-1}\right]$$

The amount of P derived from fertilizer was calculated according to Eq. 4 (Dorahy et al. 2007):

4 $$\mathrm{P\; derived \;from \;fertilizer }\;\left[\mathrm{mg\; P }\;{{\mathrm{plant}}}^{-1}\right]=\frac{{}^{33}\mathrm{P \;uptake \;plant }\left[\mathrm{kBq }\;{{\mathrm{plant}}}^{-1}\right]}{{\mathrm{S}}.\mathrm{A \;of \;labeled \;fertilizer }\;\left[\mathrm{kBq }\;{}^{33}\mathrm{P\; mg }\;{{\mathrm{P}}}^{-1}\right]}$$

whereas the quantity derived from native soil-P was calculated by subtracting the amount of P derived from fertilizer from the plant P uptake. The proportion of the total plant P derived from fertilizer (%; PdfF) was calculated by the isotopic dilution principle according to IAEA (2001) in Eq. 5

5 $${\mathrm{PdfF}} \;\, \left[\%\right]=\frac{\mathrm{S.A \;in\; plant\; sample }\;\left[{\mathrm{kBq}} \; {\mathrm{mg}}^{-1}\right] \, }{\mathrm{S.A \;of \;labeled \;fertilizer }\left[{{\mathrm{kBq}} \; {}^{33}{\mathrm{P}} \, {\mathrm{mg}}\;\mathrm{ P}}^{-1}\right]}\cdot 100$$

The proportion of P derived from native soil-P (%; PdfS) was calculated by dividing the amount of P derived from soil by the total P uptake. The efficiency of a plant to use applied fertilizer-P (PFUE) (%) was calculated according to Mohanty et al. (2006):

6 $$\mathrm{PFUE }\left[\%\right]=\frac{\mathrm{P \;derived\; from \;fertilizer} \, \left[{\mathrm{mg \;P \;p}{\mathrm{lant}}}^{-1}\right]}{{\text{Fertilizer-P}}{\mathrm{}}\;\mathrm{ applied }\;\left[{\mathrm{mg\; P \;pot}}^{-1}\right]}\cdot 100$$

The efficiencies to acquire different P sources (fertilizer-P *vs.* native soil-P) were calculated according to Wissuwa et al. (2020) in Eq. 7:

7 $${\mathrm{PAE}}\boldsymbol{ }\left[\mathrm{mg }\;\mathrm{P \;uptake}\;\boldsymbol{ }{\mathrm{g \;root \;dry \;weight}}^{-1}\right]=\frac{\mathrm{Total \;P \;uptake}\boldsymbol{ }\left[{\mathrm{mg \;P }{\;\mathrm{plant}}}^{-1}\right]}{\mathrm{Root\; dry \;weight}\boldsymbol{ }\left[{\mathrm{g }{\;\mathrm{plant}}}^{-1}\right]}\cdot 100$$

The internal efficiency to utilize P for biomass production was calculated according to Rose and Wissuwa (2012):

8 $${\mathrm{PUE}} \, \left[\mathrm{g \;DW \;m}{\mathrm{g \;P}}^{-1}\right]=\frac{\mathrm{Plant \;dry \;weight} \, \left[{{\mathrm{g}} \, {\mathrm{plant}}}^{-1}\right]}{\mathrm{Plant\; P\; content} \, \left[{\mathrm{mg \;P }{\;\mathrm{plant}}}^{-1}\right]}\cdot 100$$

### Statistical Analysis

All data were analyzed using a two-way analysis of variance (ANOVA) with the factors Genotype (GT), P-treatment (PTR), and their interactions (GT × PTR). Prior to statistical analysis, the normal distribution and variance homogeneity of the residuals were tested using the Shapiro–Wilk and Levene-test, and the R `car´ package respectively. Due to unequal error variances resulting from much higher means in the high-P treatment compared to the low-P treatment, the assumptions for variance homogeneity were violated, so separate one-way ANOVA analyses were performed for each P treatment (low and high). The statistical software R (version 4.1.3, R Core Team, USA) was used for data analysis. Genotype and P treatment means separations were done using Tukey´s honestly significant difference (HSD) test with a threshold of p ≤ 0.05 being considered significant. All presented results are reported as means ± standard error of means (± SE). Unless otherwise stated, the results presented are from one-way ANOVA analysis.

## Results

### Sorption of Fertilizer-P to the Soil Matrix

The chemical properties of the experimental soil are summarized in Table 1. The sorption experiment showed that 99.13 ± 0.05% and 99.96 ± 0.01% (high- and low-P treatment) of the ^33^P was removed from the solution by sorption to the soil matrix 60 min after the P fertilizer addition. In comparison, minimal fertilizer-P sorption was observed in the quartz sand (0.196% and 1.326% under high- and low-P conditions, respectively). In the sandy soil the sorption increased to approximately 50.39% under low-P conditions and 10.25% under high-P conditions.

### Plant Responses 9 days After Emergence

At the initial harvest (9 DAE – two leaf stage) the P treatment had no significant effect on plant biomass while a significant increase in shoot P content was found in the high-P treatment compared to the low-P treatment (Suppl. Table S2). In the low-P treatment AB199 showed a significant higher shoot dry weight than Nerica4 while DJ123 and AB67 did not differ significantly from any of the genotypes (suppl. Table S3). In the high-P treatment, AB199 and DJ123 had a significantly higher shoot dry weight than Nerica4 while AB67 did not differ significantly from any of the genotypes (suppl. Table S3). Analysis of the seed-P content showed significant differences between the genotypes, with AB199 having the highest and DJ123 having the lowest seed-P content while AB67 and Nerica4 were not significantly different to either of them (suppl. Table S4). Assuming that 70% of seed-P had been transferred to the plant within the 9 DAE growth period (Julia et al. 2018), we estimated total P uptake to average 43 µg plant^−1^ across both P treatments (Suppl. Table S2). The PAE differed significantly between the P treatments and was 43% higher in the high-P treatment compared to the low-P treatment (suppl. Fig. S1). Moreover, DJ123 showed a 97% higher PAE relative to Nerica4, whereas AB67 and AB199 did not differ significantly from any of the genotypes under low-P conditions. In the high-P treatment no significant differences were found in total P uptake (Suppl. Table S3) and PAE between the genotypes (suppl. Fig. S1).

### Plant Responses 34 Days After Emergence

At the second harvest (34 DAE—beginning of tillering) the shoot dry weight increased 2.4-fold from the low-P to the high-P treatment (Table 2). Significant genotype variations were observed in both P treatments. DJ123 exhibited the highest shoot dry weight among all genotypes under high-P conditions, whereas under low-P conditions, DJ123 and AB199 had similar shoot dry weights. Nerica4 had the lowest shoot dry weight overall, while AB67 had comparable shoot biomass to AB199 under low-P conditions (Table 2). Among the investigated genotypes, DJ123 showed the strongest biomass response upon P fertilization with a 3.2-fold increase in shoot dry weight, followed by Nerica4 with a 2.4-fold increase, and the AB lines (AB199—2.1-fold increase and AB67—2.0-fold increase). At 34 DAE the shoot tissue P concentration of 1.67 mg g^−1^ under high-P indicated a moderate but sufficient P supply while the tissue P concentration of 0.58 mg g^−1^ indicated that plants were severely P deficient in the low-P treatment (Yoshida 1981; Hedley et al. 1994). Significant differences were also found for the root-to-shoot ratios (R:S ratio), with Nerica4 having a 41% higher R:S ratio compared to all other genotypes, mainly due to its reduced shoot biomass (Table 2).

**Table 2:** Plant dry weight and total P uptake of the tested genotypes at the second harvest point 34 DAE

Low-P		AB199		AB67		DJ123		Nerica4	
Shoot dry weight	[mg plant ^−1^]	232.9	ab	219.9	b	249.2	a	149.4	c
		± 3.0		± 5.7		± 6.5		± 7.9	
Root dry weight	[mg plant ^−1^]	157.0	a	153.1	a	191.5	a	151.4	a
		± 13.1		± 8.6		± 10.5		± 7.1	
Root:shoot-ratio		0.68	b	0.70	b	0.77	b	1.01	a
		± 0.06		± 0.03		± 0.05		± 0.01	
Shoot P concentration	[mg P g^−1^]	0.51	c	0.52	bc	0.64	ab	0.67	a
		± 0.01		± 0.04		± 0.03		± 0.04	
Root P concentration	[mg P g^−1^]	0.56	a	0.54	a	0.69	a	0.65	a
		± 0.02		± 0.04		± 0.06		± 0.03	
Plant P content	[mg P plant^−1^]	0.206	b	0.199	b	0.294	a	0.199	b
		± 0.006		± 0.016		± 0.021		± 0.010	
Total P uptake	[mg P plant^−1^]	0.118	b	0.115	b	0.22	a	0.12	b
		± 0.008		± 0.016		± 0.02		± 0.011	
High-P									
Shoot dry weight	[mg plant ^−1^]	720.0	b	655.3	bc	1040.3	a	506.6	c
		± 34.13		± 40.99		± 42.38		± 39.16	
Root dry weight	[mg plant ^−1^]	338.2	b	316.4	b	519.4	a	296.0	b
		± 10.07		± 22.74		± 41.41		± 17.10	
Root:shoot-ratio		0.47	b	0.48	b	0.50	ab	0.59	a
		± 0.02		± 0.01		± 0.03		± 0.02	
Shoot P concentration	[mg P g^−1^]	1.61	ab	1.54	b	1.74	ab	1.79	a
		± 0.02		± 0.05		± 0.09		± 0.04	
Root P concentration	[mg P g^−1^]	1.21	a	1.11	a	1.14	a	1.25	a
		± 0.05		± 0.02		± 0.03		± 0.05	
Plant P content	[mg P plant^−1^]	1.57	b	1.36	b	2.40	a	1.27	b
		± 0.09		± 0.09		± 0.11		± 0.09	
Total P uptake	[mg P plant^−1^]	1.483	b	1.273	b	2.333	a	1.193	b
		± 0.089		0.092		± 0.111		± 0.088	

Values are means ± SE. Distinct letters indicate significant differences between genotypes (*p* < 0.05, Tukey’s HSD, *n* = 4)

**Fig. 1:**
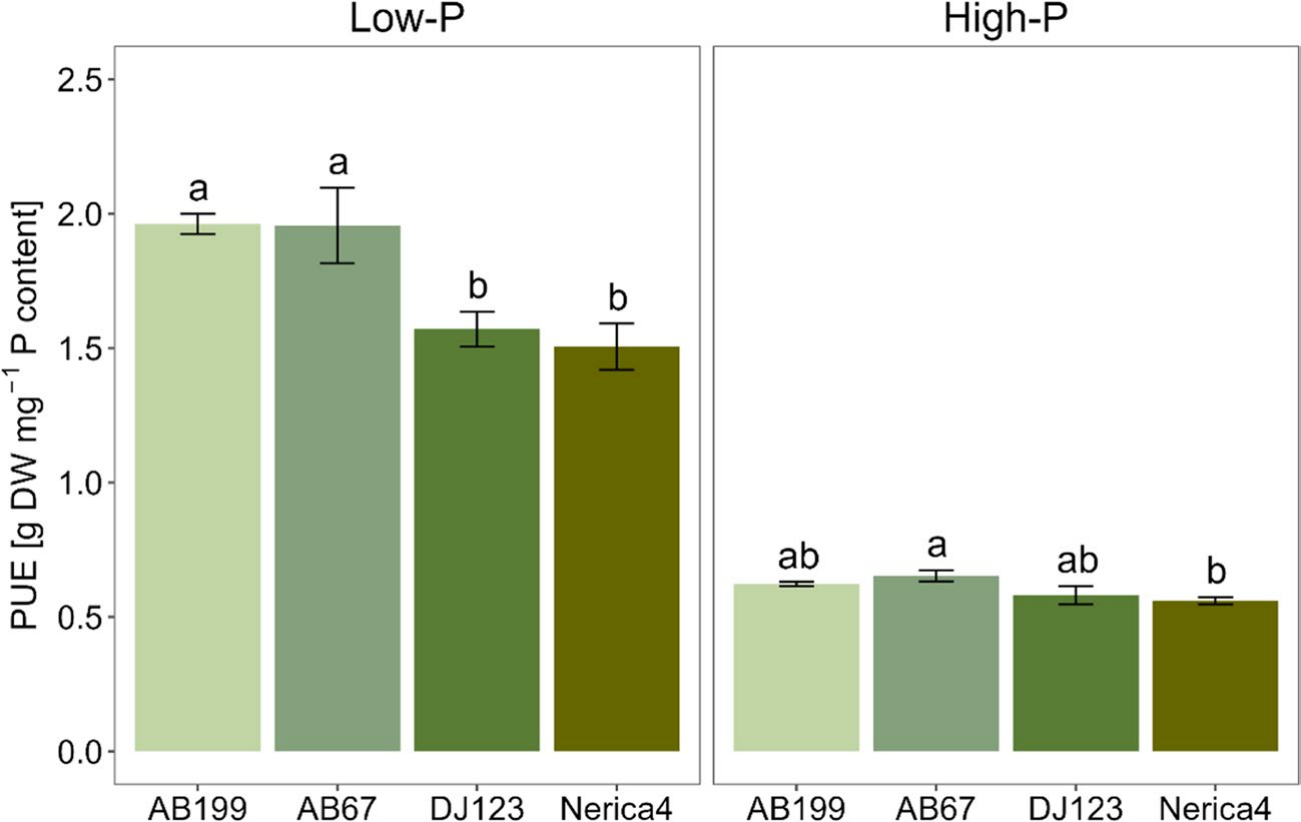
Phosphorus utilization efficiency (PUE) of the four genotypes AB199, AB67, DJ123 and Nerica4 34 days after emergence under low- and high-P conditions. Variation is given as SE, *n* = 4. Distinct letters indicate significant differences between genotypes (*p* < 0.05, Tukey’s HSD) in the low- and high-P treatment, respectively

**Fig. 2:**
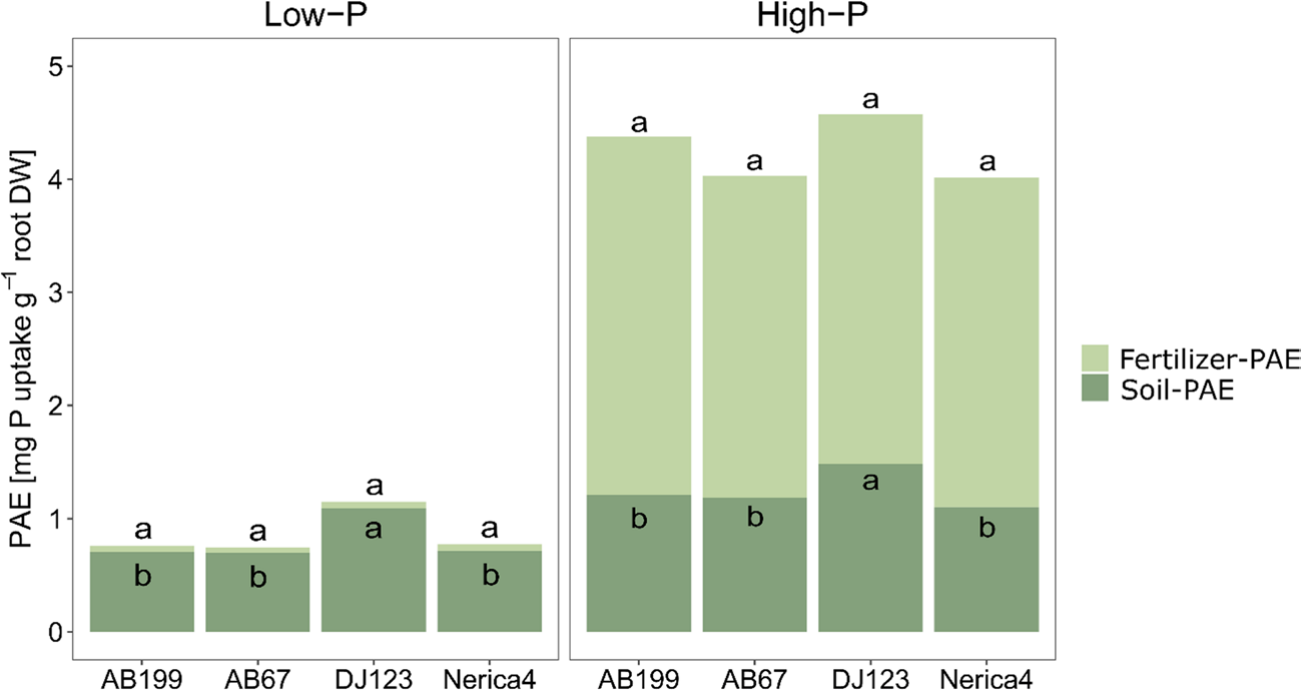
P acquisition efficiency (PAE) of native soil-P and fertilizer-P of the four genotypes AB199, AB67, DJ123 and Nerica4 at 34 days after emergence under low- and high-P conditions (*n* = 4). Distinct letters indicate significant differences between genotypes (*p* < 0.05, Tukey’s HSD) in the low- and high-P treatment, respectively

At the second harvest, the total P uptake increased tenfold from the low- to the high-P treatment (Suppl. Table S5). Significant genotypic variation in total P uptake was observed for both P supply levels at 34 DAE (Table 2). In the low-P treatment, DJ123 exhibited, on average, a 90% higher total P uptake compared to all the other genotypes (*p* < 0.05), while root biomass was similar among all genotypes (*p* > 0.05). While the other genotypes showed no significant differences in terms of total P uptake (Table 2), the sister lines AB67 and AB199 exhibited significantly higher internal PUE compared to Nerica4 and DJ123 (Fig. 1). However, DJ123 showed a significantly higher PAE in the low-P treatment compared to all other genotypes (Fig. 2). In the high-P treatment, DJ123 showed a 77% higher total P uptake (*p* < 0.05) compared to the other genotypes, accompanied by an average 64% increase in root dry weight relative to the other genotypes. No differences in PAE were observed among the genotypes under high-P conditions. In the high-P treatment AB67 had a significantly higher internal PUE than Nerica4, while AB199 and DJ123 did not differ significantly from either of them (Fig. 1).

### Utilization of Different P Sources

**Fig. 3:**
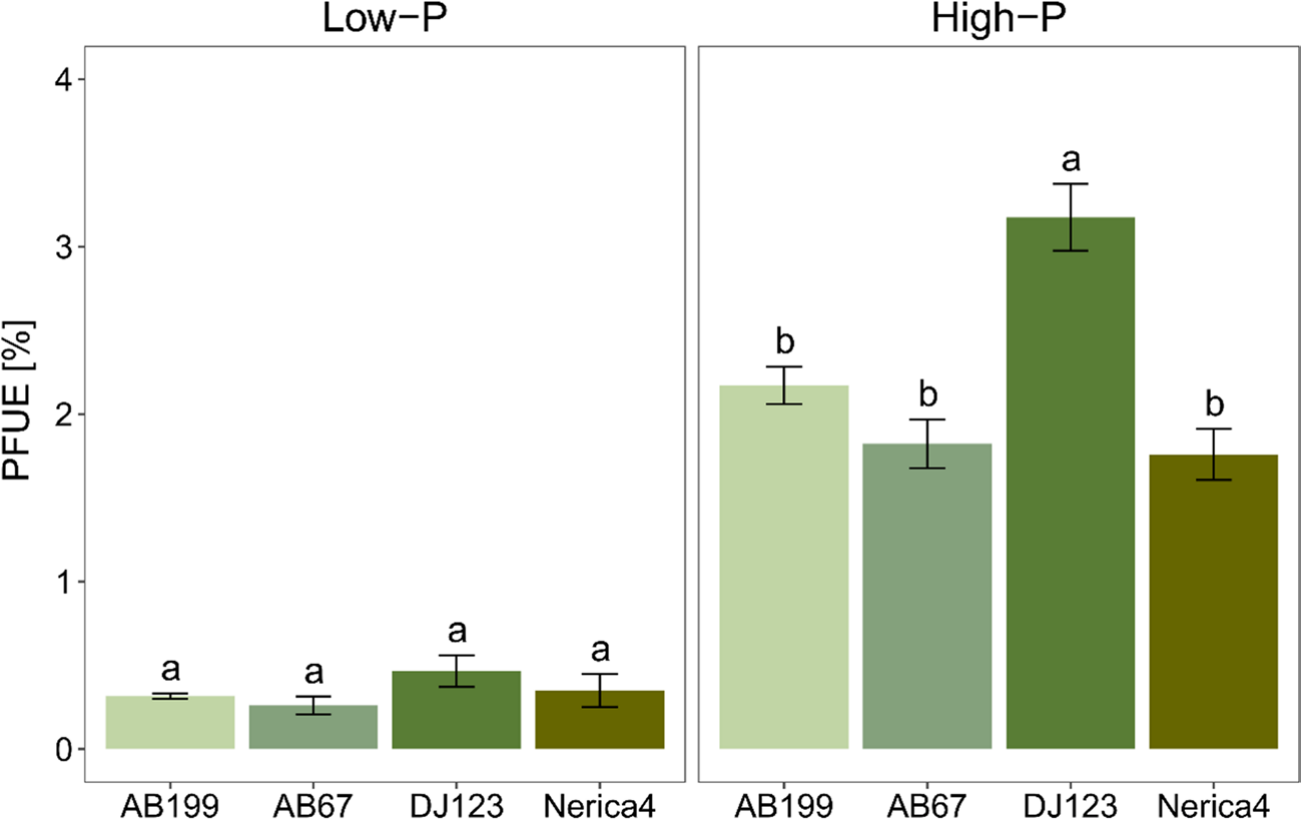
Applied P use efficiency (PFUE) of the four genotypes AB199, AB67, DJ123 and Nerica4 34 days after emergence under low- and high-P conditions. Variation is given as SE, *n* = 4. Distinct letters indicate significant differences between genotypes (*p* < 0.05, Tukey’s HSD) in the low- and high-P treatment, respectively

At 34 DAE, the applied PFUE was, on average, 4 times higher in the high-P treatment compared to the low-P treatment. Figure 3 depicts that the genotypes followed a similar trend in PFUE at each P level. However, significant differences were observed in the high-P treatment only, where DJ123 exhibited a significantly higher applied PFUE compared to the other genotypes.

**Fig. 4:**
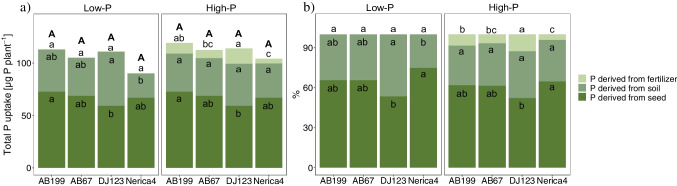
**a**.) Quantity and **b**.) percent of total plant P derived from seed-P, native soil-P and fertilizer-P of the four genotypes AB199, AB67, DJ123 and Nerica4 at 9 days after emergence under low- and high-P conditions (*n* = 4). Distinct letters indicate significant differences between genotypes (*p* < 0.05, Tukey’s HSD) in the low- and high-P treatment, respectively. Distinct capital letters indicate significant differences in total plant P content. For total P uptake and % P derived from fertilizer under low-P conditions the values were so low, that no bars are visible

In this study, the uptake of P was categorized into P derived from the seed, fertilizer, or from the native soil-P pool. This categorization was based on the application of ^33^P-labeled fertilizer and the measurement of P content in seeds and in the whole plant. At the first harvest (9 DAE), it was estimated that on average, 65% and 60% of total P was derived from seed-P under low- and high-P conditions, respectively (Fig. 4b). After 34 DAE, seed-P was assumed to contribute 38.1% and 5.4% to total plant P in the low-P and high-P treatments, respectively (suppl. Table S5).

**Fig. 5:**
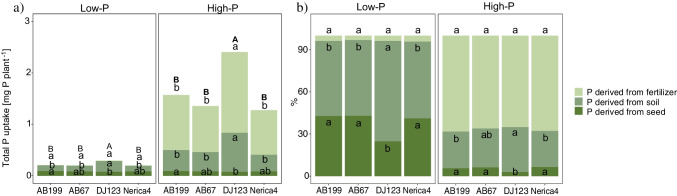
**a**.) Quantity and **b**.) percent of total plant P derived from seed-P, native soil-P and fertilizer-P of the four genotypes AB199, AB67, DJ123 and Nerica4 at 34 days after emergence under low- and high-P conditions. Error bars indicate ± SE, *n* = 4. Distinct letters above the bars indicate significant differences between genotypes (*p* < 0.05, Tukey’s HSD) in the low- and high-P treatment, respectively. Distinct capital letters indicate significant differences in total plant P content. For total P uptake derived from fertilizer under low-P conditions the values were so low, that no bars are visible

For the first harvest, the uptake and contribution of fertilizer-P to total plant P ranged from 0.12% to 8.01% in the low-P and high-P treatments, respectively (Fig. 4b). At 34 DAE, the amount of fertilizer-P acquired was 127 times higher under high-P conditions compared to low-P conditions (suppl. Table S5). Genotypic differences were only observed under high-P conditions, with DJ123 exhibiting significantly higher uptake of fertilizer-P compared to all the other genotypes (Fig. 5a). However, when considering the relative contribution of P derived from fertilizer to total P, no significant genotypic differences were evident (Fig. 5b).

At the second harvest, plants grown in the high-P soil acquired approximately 333 µg more native soil-P compared to plants grown in the low-P soil (suppl. Table S5). As depicted in Table 3, the P treatment had the greatest impact (69.4%) on the amount of native soil-P taken up by the plants. On a relative basis, native soil-P contributed more to the total plant P under low-P conditions (58.2%) compared to high-P conditions (27.8%) (Fig. 5b). As shown in Fig. 5a, there were significant genotypic differences observed in both P treatments. DJ123 exhibited a 93% and 107% higher uptake of native soil-P (both *p* < 0.05) compared to the other genotypes in the low-P and high-P treatments, respectively. The relative contribution of native soil-P to total plant P was highest for DJ123 (71%) while the other genotypes ranged between 53 and 55% under P-deficient conditions (Fig. 5b). Furthermore, when additional P was supplied, DJ123 showed the highest percentage of P derived from native soil-P compared to Nerica4 and AB199, while AB67 did not differ significantly from any of the genotypes (Fig. 5b).

**Table 3:** Two-way fixed-effects ANOVA results using quantity of plant P derived from soil as the response variable

Predictor	*df*	Sum of Squares	Mean Square	*F* value	*P*-value	Variance component [%]
GT	3	0.3749	0.125	69.15	5.92^–12^	14.7
PTR	1	0.8897	0.8897	492.31	< 2^–16^	69.4
GT:PTR	3	0.1356	0.0452	25.01	1.46^–07^	16.0
Residuals	24	0.0434	0.0018			

*df*, degrees of freedom; GT, genotype; PTR, P-treatment and GT:PTR the interaction of both of them. Variance components were calculated according to Rasch et al. (1983)

### P Acquisition Efficiency According to P Sources (Fertilizer-P vs. Native Soil-P)

At the second harvest, the fertilizer-P acquisition efficiency (fertilizer-PAE) was substantially lower than the native soil-P acquisition efficiency (soil-PAE) under low-P conditions. However, under high-P conditions, more P was taken up from fertilizer per gram of root dry weight compared to P obtained from the soil (Fig. 2).

The fertilizer-PAE was 56-fold greater in the high-P treatment than in the low-P treatment, with no significant genotypic differences. Considering the soil-PAE, a 55% increase was observed for the high-P treatment compared to the low-P treatment. The genotypic variation of soil-PAE followed a similar trend in each P level, where DJ123 exhibited significantly higher soil-PAE than all the other genotypes, while the AB lines and Nerica4 did not differ significantly from each other. Therefore, DJ123 had an average 54% and 27% greater soil-PAE than the other genotypes in the low- and high-P treatment, respectively.

## Discussion

Efficient P fertilizer use in crops enables cost-effective production, reduces fertilizer expenses for farmers, minimizes nutrient runoff into water bodies and promotes sustainable agricultural practices (Fixen and Garcia 2015). However, the use of freshly applied fertilizer-P is generally low in highly P-fixing soils (Otani and Ae 1996; Oo et al. 2020), which is due to the strong P fixation resulting from high amounts of active Al- and Fe- (hydr-)oxides and amorphous silicates protecting P from mobilization (Otani and Ae 1996; Holford 1997; Nishigaki et al. 2019). The Andosol used in our study had high concentrations of ammonium oxalate extractable Al and Fe originating from poorly crystalline soil minerals and nearly all applied fertilizer was removed from the solution after one hour of incubation (99.13% and 99.96% under high- and low-P conditions). The high fertilizer-P sorption observed in the Andosol during the sorption experiment, particularly when compared to a sandy soil, underlines two key points: (1) the importance of native soil-P resources for plant P uptake in environments with high P fixation, as demonstrated in the latter, and (2) the overall ability of plants to partially overcome P sorption and acquire highly sorbed fertilizer-P. The plants ability to acquire highly sorbed fertilizer-P, was already evident at the 2-leaf stage (9 DAE), where the cultivars were able to use 0.01% and 0.04% of the applied fertilizer P under low- and high-P conditions, respectively. A higher P utilization was recorded by Julia et al. (2018), who observed that 80% of exogenously applied P was taken up 6–8 d after germination by the rice genotype IR64 grown in nutrient solution and concluded that P uptake by rice started before the seedlings reached autotrophy. At the beginning of tillering (34 DAE), on average 2.2% of the applied fertilizer was used in the high-P treatment, whereas less than 0.5% was used in the low-P treatment, confirming the low plant P availability in this soil. However, with a growth period of 90–120 days for upland rice till harvest and considering the decline of the relative growth rate with a concomitant increase of P remobilization from senescing tissues (Veneklaas et al. 2012), it is reasonable to estimate that the uptake of fertilizer-P at harvest in the high-P treatment would be around 10–20%. This reflects a relatively low, but common level of applied PFUE on the tested Andosol (Otani and Ae 1996).

Apart from fertilizer-P, another source of P for the plant is the seed-P and its utilization is a crucial factor, particular in low-P soils, where it constitutes a significant part of the plant P supply during early growth stages (White and Veneklaas 2012). At 9 DAE, plants derived a substantial portion of their P content from seed reserves. On average 65% and 60% of the plant P originated from seed-P under low- and high P conditions, respectively. This suggests that exogenous P uptake from the soil only contributed 35% or 40% of plant P at this early stage. However, caution is warranted when interpreting these results regarding native soil-P uptake at 9 DAE. Our calculations for seed-P mobilization rely on assumptions and uncertainties remain regarding the actual seed-P mobilization. At later growth stages, seed-P normally becomes depleted, forcing plants to rely on root P uptake from the soil matrix (Veneklaas et al. 2012). We estimated that seed-P contributed 38% to the total plant P in the low- and 5% in the high-P treatment, at the second harvest. This highlights the significant role of seed-P in supporting plant P nutrition even at tillering under low P conditions. Therefore, considering seed-P contribution becomes crucial when assessing total P uptake in the early plant developmental stages under P limiting conditions.

As plant development progresses, the uptake of exogenous P from the soil matrix becomes increasingly important. This P uptake varies between genotypes and is strongly influenced by the soil P status (Rose et al. 2012; Pariasca-Tanaka et al. 2015). Previous research has demonstrated that DJ123 exhibits superior P uptake ability in low-P soils compared to the widely grown variety Nerica4 (Koide et al. 2013; Mori et al. 2016; Wissuwa et al. 2020) and this was confirmed by our results. Furthermore, AB199 outperformed Nerica4 in biomass production under low-P conditions, indicating that AB199 may be a promising genotype for breeding programs targeting low input systems. The weak performance of Nerica4 under low-P conditions has been shown previously (Koide et al. 2013; Mori et al. 2016; Wissuwa et al. 2020). However, Nerica4 is commonly considered as a high yielding rice variety (Somado et al. 2008; Moukoumbi et al. 2015) with a good responsiveness to fertilizer application and a sufficient tolerance to drought on more fertile soils (Wissuwa et al. 2020). In contrast to these findings, in our study Nerica4 did not show a strong positive response to fertilizer-P application at tillering, when compared to other genotypes. This may be due to the fact that Nerica4 tends to have higher growth rates than DJ123 at later stages of development (Matthias Wissuwa, personal communication). In addition to P uptake, the assessment of the efficiency at which P is acquired is critical for identifying superior upland rice genotypes for breeding purposes. In this regard, DJ123 showed superior PAE compared to the other genotypes under low-P conditions which is in accordance with previous studies (Mori et al. 2016; Wissuwa et al. 2020). However, Shimamura et al. (2021) found no increased PAE of DJ123 21 days after sowing and accounted this to a decreased acidity tolerance relative to other tested genotypes. Furthermore, Rakotoson et al. (2020) found no increased PAE which was, however, probably caused by restricted root growth conditions in a rhizobox setup. The significantly higher root dry weight and increased total P uptake of DJ123 in the high-P treatment were accompanied by similar PAE among all genotypes, which aligns with previous findings (Wissuwa et al. 2020).

Investigating how different P sources contribute to P uptake and their efficiency of acquisition provides valuable insights into plant adaptive strategies. With reduced fertilizer application, native soil-P made the greatest contribution to total plant P content in our study. Consequently, under low-P conditions no genotypic differences in the contribution of fertilizer-P to total P uptake were observed. Thus, differences in total P uptake among the genotypes can be attributed to differences in native soil-P uptake, with DJ123 exhibiting significantly higher uptake of native soil-P compared to all other genotypes. Furthermore, when considering the efficiency of the root to acquire P under low-P conditions, DJ123 showed a significantly higher native soil-PAE in comparison to the AB lines and Nerica4 while the fertilizer-PAE was not significantly different between the genotypes. This suggests that the increased PAE in DJ123 under low-P conditions can be associated with mechanisms involved in mobilizing P from native soil-P sources. These mechanisms may involve different soil–plant processes including pH changes in the rhizosphere, the exudation of P solubilizing compounds such as organic anions or phenolics, the exudation of phosphatases or the symbiosis with mycorrhiza (Marschner and Römheld 1994; George et al. 2006; Landi et al. 2006; Kuppe et al. 2022). Additionally, plants might be able to support a certain rhizosphere microbiota which enhances P mobilization (Oliveira et al. 2009). Native soil-P sources comprise a wide range of different chemical P forms and genotypes might differ in their ability to assess certain P forms. For example, rice (rice cultivar X265) grown on highly weathered soils mainly accessed the labile organic and inorganic P pools (resin-P and NaHCO_3_-Po; Nishigaki et al. (2019)), whereas upland rice cultivars (Azucena and Dinorado) mainly accessed P from NaOH extractable pools (Hedley et al. 1994). The mobilization of organically bound P sources has received some attention (George et al. 2018; Nishigaki et al. 2019), though so far no evidence has been found to suggest that rice genotypes differ in their ability to access organic P (Rakotoson et al. 2020; Shimamura et al. 2021). In contrast to wheat, rice grown on a low-humic Andosol was able to acquire also residual-P, which would be indicative for a high capability of rice to mobilize recalcitrant fertilizer-P forms (Takahashi 2007). Furthermore, plant morphological adaptations can contribute to P uptake, with DJ123 having a higher proportion of fine roots and a greater root hair length compared to other genotypes as shown in previous studies (Nestler and Wissuwa 2016; Wissuwa et al. 2020). Our results indicate that upland rice genotypes, varying in their PAE, differ in their ability to access sparingly available native soil-P forms under low-P conditions. However, our experimental setup did not allow to differentiate between native soil-P forms. Therefore, further studies focusing on genotypic differences in PAE and the identification of specific soil-P pools that are accessed by P efficient genotypes are needed and would allow for more precise breeding attempts for increased PAE in rice.

Our study demonstrated a positive relation between fertilizer application rates, and plant uptake of both, fertilizer-derived P uptake but also native soil-P. This observation aligns with findings in *Lolium perenne* where P fertilizer addition in soils with high P availability, resulted in a suppression of native soil-P uptake, while the addition of fertilizer-P resulted in an increase in the uptake of native soil-P in soils with low P availability (Morel and Fardeau 1989, 1990). Considering the limited availability of P in highly P-fixing soils, P fertilization can therefore play a crucial role in promoting the utilization of native soil-P resources, particularly in low-input systems. Furthermore, significant genotypic differences were observed in the uptake of both native soil-P and fertilizer-P, with DJ123 exhibiting significantly higher uptake of native soil-P compared to the other tested genotypes. While the greater root dry weight in DJ123 can explain the increased uptake of fertilizer-P (fertilizer-PAE, *p* > 0.05), the enhanced uptake of native soil-P can be attributed to a higher PAE in DJ123. These results indicate that even under high-P conditions, DJ123 employed specific mechanisms that enable this genotype to efficiently acquire native soil-P, contributing to its superior performance. Further investigations are needed to elucidate the underlying factors responsible for the higher native soil-PAE in DJ123 and its implications for upland rice production in low-input systems. To gain a more comprehensive understanding of genotype-P fertilizer interaction, further studies incorporating a broader range of genotypes are warranted. These studies should encompass later growth stages to elucidate whether genotypes that are performing well under high-P conditions (i.e. Nerica4) exhibit enhanced fertilizer-P uptake capacities at these later growth stages.

Besides PAE the internal PUE is of great importance for enhancing yields under conditions of low soil-P availability (Wang et al. 2010a; Rose and Wissuwa 2012). However, due to the poor uptake efficiency of fertilizer-P, previous efforts to enhance P efficiency in upland rice through plant breeding have primarily focused on improving PAE (Rose et al. 2011). Screening for genotypes with differential PUE poses a challenge in breeding, particularly when high PAE in plants is often associated with low PUE (Rose et al. 2011; Mori et al. 2016; Vandamme et al. 2016). In our experiment this was the case for DJ123, which exhibited the highest PAE among all tested genotypes but had a comparably low PUE. For DJ123, the low PUE under low-P conditions was primarily due to increased total P uptake and biomass production while maintaining high tissue P concentrations. On the other hand, Nerica4, being inefficient in acquiring P from the soil, likely experienced greater P deficiency stress and consequently was unable to utilize the available P for increased biomass production. Rose et al. (2016) suggested to compare genotypes at equal shoot P content in soil-based experiments when screening for PUE. At as average shoot P content of 0.111 mg P per plant in the low-P treatment, the AB lines displayed, a 25% higher PUE compared to Nerica4. This suggests that the AB lines were able to achieve greater biomass production with less P, indicating an increased tolerance to P stress compared to Nerica4. Therefore, AB199 and AB67 could be interesting donors for improved PUE under low-P conditions.

## Conclusions

Our findings demonstrate that the P acquisition efficiency of upland rice during the early growth stage is intricately linked to its ability to extract P from the native soil-P pool, regardless of low- or high-P conditions. DJ123 stands out as particularly adept at acquiring native soil-P. Interestingly, under increased P fertilization, all genotypes intensified uptake from both native soil-P and fertilizer-P, with DJ123 maintaining a distinct advantage in accessing native soil-P. This highlights the importance of adequate P fertilization for optimal utilization of native soil-P resources. Moreover, genotypic differences in P acquisition efficiency and the contribution of native soil-P underscore the need for further studies on specific soil-P pools accessed by efficient genotypes, allowing more targeted breeding for enhanced P acquisition efficiency in upland rice. Our findings emphasize the complex interplay between P sources and genotypic responses, offering insights for sustainable rice cultivation in diverse P conditions.

## Supplementary Information

Below is the link to the electronic supplementary material.

Supplementary file1 (DOCX 90 KB)

## Data Availability

The data that support the findings of this study are available on request from the corresponding author, EM.

## References

[CR1] Adesemoye AO, Kloepper JW (2009) Plant – microbes interactions in enhanced fertilizer-use efficiency. Appl Microbiol Biotechnol 85:1–12. 10.1007/s00253-009-2196-0 10.1007/s00253-009-2196-0 19707753

[CR2] Aerts R (1996) Nutrient resorption from senescing leaves of perennials: are there general patterns? J Ecol 84:597–608. 10.2307/2261481

[CR3] Africa Rice Center (WARDA)/FAO/SAA (2008) NERICA: the New Rice for Africa – a Compendium. In: Somado EA, Guei RG, Keya SO (eds) Cotonou, Benin: Africa Rice Center (WARDA); Rome, Italy: FAO; Tokyo, Japan: Sasakawa Africa Association, p 210

[CR4] Atakora WK, Fosu M, Abebrese SO, Asanta M, Wissuwa M (2015) Evaluation of low phosphorus tolerance of rice varieties in northern Ghana. Sust Agric Res 4:109–114. 10.5539/sar.v4n4p109

[CR5] Balemi T, Negisho K (2012) Management of soil phosphorus and plant adaptation mechanisms to phosphorus stress for sustainable crop production: A review. J Soil Sci Plant Nutr 12:547–561. 10.4067/s0718-95162012005000015

[CR6] Bray RH, Kurtz LT (1945) Determination of total organic and available forms of phosphorus in soils. Soil Sci 59:39–45. 10.1097/00010694-194501000-00006

[CR7] Chen Z, Wang L, Cardoso JA et al (2023) Improving phosphorus acquisition efficiency through modification of root growth responses to phosphate starvation in legumes. Front Plant Sci 14:1–15. 10.3389/fpls.2023.1094157 10.3389/fpls.2023.1094157 PMC9950756 36844096

[CR8] Dorahy CG, Blair GJ, Rochester IJ, Till AR (2007) Availability of P from 32P-labelled endogenous soil P and 33P-labelled fertilizer in an alkaline soil producing cotton in Australia. Soil Use Manag 23:192–199. 10.1111/j.1475-2743.2007.00083.x

[CR9] Fixen PE, Brentrup F, Bruulsema T, Garcia F, Norton R, Zingore S (2015) Nutrient/fertilizer use efficiency: measurement, current situation and trends. In: Drechsel P, Heffer P, Magen H, Mikkelsen R, Wichelns D (eds) Managing water and fertilizer for sustainable agricultural intensification. IFA, IWMI, IPNI, IPI. First edition, Paris, France, pp 8–37

[CR10] George TS, Turner BL, Gregory PJ et al (2006) Depletion of organic phosphorus from Oxisols in relation to phosphatase activities in the rhizosphere. Eur J Soil Sci 57:47–57. 10.1111/j.1365-2389.2006.00767.x

[CR11] George TS, Giles CD, Menezes-Blackburn D et al (2018) Organic phosphorus in the terrestrial environment: a perspective on the state of the art and future priorities. Plant Soil 427:191–208. 10.1007/s11104-017-3391-x

[CR12] Grant CA, Flaten DN, Tomasiewicz DJ, Sheppard SC (2001) The importance of early season phosphorus nutrition. Can J Plant Sci 81:211–224

[CR13] Grant C, Bittman S, Montreal M et al (2005) Soil and fertilizer phosphorus: Effects on plant P supply and mycorrhizal development. Can J Plant Sci 85:3–14

[CR14] Hashimoto Y, Kang J, Matsuyama N, Saigusa M (2012) Path analysis of phosphorus retention capacity in allophanic and non-allophanic Andisols. Soil Sci Soc Am J 76:441–448. 10.2136/sssaj2011.0196

[CR15] Hedley MJ, Kirk GJD, Santos MB (1994) Phosphorus efficiency and the forms of soil phosphorus utilized by upland rice cultivars. Plant and Soil 158:53–62

[CR16] Holford ICR (1997) Soil phosphorus: its measurement, and its uptake by plants. Soil Res 35:227–240. 10.1071/S96047

[CR17] IAEA (2001) Use of isotope and radiation methods in soil and water management and crop nutrition. International Atomic Energy Agency, Vienna, Austria, p 247

[CR18] Julia CC, Rose TJ, Pariasca-Tanaka J et al (2018) Phosphorus uptake commences at the earliest stages of seedling development in rice. J Exp Bot 69:5233–5240. 10.1093/jxb/ery267 30053197 10.1093/jxb/ery267

[CR19] Koide Y, Pariasca Tanaka J, Rose T et al (2013) Qtls for phosphorus deficiency tolerance detected in upland nerica varieties. Plant Breed 132:259–265. 10.1111/PBR.12052

[CR20] König N (2005) Handbuch forstliche analytik (HFA) – Grundwerk. Eine Loseblatt-Sammlung der Analysemethoden im Forstbereich. Chapter A3. Published by Gutachterausschuss forstliche analytik (GFA). German federal ministry of food and agriculture (BMEL)

[CR21] Kuppe CW, Kirk GJD, Wissuwa M, Postma JA (2022) Rice increases phosphorus uptake in strongly sorbing soils by intra-root facilitation. Plant Cell Environ 45:884–899. 10.1111/pce.14285 35137976 10.1111/pce.14285

[CR22] Landi L, Valori F, Ascher J et al (2006) Root exudate effects on the bacterial communities, CO2 evolution, nitrogen transformations and ATP content of rhizosphere and bulk soils. Soil Biol Biochem 38:509–516. 10.1016/j.soilbio.2005.05.021

[CR23] Marschner H, Römheld V (1994) Strategies of plants for acquisition of iron. Plant Soil 165:261–274. 10.1007/BF00008069

[CR24] Mohanty S, Paikaray NK, Rajan AR (2006) Availability and uptake of phosphorus from organic manures in groundnut (*Arachis hypogea* L.)–corn (*Zea mays* L.) sequence using radio tracer technique. Geoderma 133:225–230. 10.1016/J.GEODERMA.2005.07.009

[CR25] Morel C, Fardeau JC (1989) Native soil and fresh fertilizer phosphorus uptake as affected by rate of application and P fertilizers. Plant Soil 115:123–128. 10.1007/BF02220702

[CR26] Morel C, Fardeau JC (1990) Uptake of phosphate from soils and fertilizers as affected by soil P availability and solubility of phosphorus fertilizers. Plant Soil 121:217–224. 10.1007/BF00012315

[CR27] Mori A, Fukuda T, Vejchasarn P et al (2016) The role of root size versus root efficiency in phosphorus acquisition in rice. J Exp Bot 67:1179–1189. 10.1093/jxb/erv557 26842979 10.1093/jxb/erv557

[CR28] Moukoumbi YD, Kolade O, Drame KN et al (2015) Genetic relationships between interspecific lines derived from *Oryza glaberrima* and *Oryza sativa* crosses using microsatellites and agro-morphological markers. Spanish J Agric Res 13(2). 10.5424/sjar/2015132-6330

[CR29] Murphy J, Riley RP (1962) A modified single solution method for the evaluation of phosphate in natural water. Anal Chim Acta 27:31–36

[CR30] Nanzyo M (2002) Unique properties of volcanic ash soils. Glob J Environ Res 6:99–112

[CR31] Nestler J, Wissuwa M (2016) Superior root hair formation confers root efficiency in some, but not all, rice genotypes upon P deficiency. Front Plant Sci 7:1935. 10.3389/fpls.2016.01935 10.3389/fpls.2016.01935 PMC5174101 28066487

[CR32] Nishigaki T, Tsujimoto Y, Rinasoa S et al (2019) Phosphorus uptake of rice plants is affected by phosphorus forms and physicochemical properties of tropical weathered soils. Plant Soil 435:27–38. 10.1007/s11104-018-3869-1

[CR33] Oliveira CA de, Marriel IE, Gomes EA et al (2009) Bacterial diversity in the rhizosphere of maize genotypes contrasting for phosphorus use efficiency. Pesqui Agrop Bras 44:1473–1482. 10.1590/S0100-204X2009001100015

[CR34] Oo AZ, Tsujimoto Y, Rakotoarisoa NM et al (2020) P-dipping of rice seedlings increases applied P use efficiency in high P-fixing soils. Sci Rep 10:1–10. 10.1038/s41598-020-68977-1 32681148 10.1038/s41598-020-68977-1 PMC7368074

[CR35] Otani T, Ae N (1996) Phosphorus (P) uptake mechanisms of crops grown in soils with low P status: I. Screening of crops for efficient P uptake. Soil Sci Plant Nutr 42:155–163. 10.1080/00380768.1996.10414699

[CR36] Otani T, Ae N (1999) Extraction of organic phosphorus in Andosols by various methods. Soil Sci Plant Nutr 45:151–161. 10.1080/00380768.1999.10409331

[CR37] Pariasca-Tanaka J, Vandamme E, Mori A et al (2015) Does reducing seed-P concentrations affect seedling vigor and grain yield of rice? Plant Soil 392:253–266. 10.1007/s11104-015-2460-2

[CR38] Rakotoson T, Holz M, Wissuwa M (2020) Phosphorus deficiency tolerance in *Oryza sativa*: Root and rhizosphere traits. Rhizosphere 14:100198. 10.1016/j.rhisph.2020.100198

[CR39] Ranaivo HN, Lam DT, Ueda Y et al (2022) QTL mapping for early root and shoot vigor of upland rice (*Oryza** sativa* L.) under P deficient field conditions in Japan and Madagascar. Front Plant Sci 13:4189. 10.3389/FPLS.2022.1017419/BIBTEX 10.3389/fpls.2022.1017419 PMC9637880 36352889

[CR40] Rasch D, Bock J, Dörfel H et al (1983) Einführung in die Biostatistik. VEB Deutscher Landwirtschaftsverlag, p 276

[CR41] Richardson AE, Hocking PJ, Simpson RJ, George TS (2009) Plant mechanisms to optimise access to soil phosphorus. Crop Pasture Sci 60:124. 10.1071/CP07125

[CR42] Richardson AE, Lynch JP, Ryan PR et al (2011) Plant and microbial strategies to improve the phosphorus efficiency of agriculture. Plant Soil 349:121–156. 10.1007/s11104-011-0950-4

[CR43] Rose TJ, Pariasca-Tanaka J, Rose MT et al (2012) Seeds of doubt: Re-assessing the impact of grain P concentrations on seedling vigor. J Plant Nutr Soil Sci 175:799–804. 10.1002/jpln.201200140

[CR44] Rose TJ, Mori A, Julia CC, Wissuwa M (2016) Screening for internal phosphorus utilisation efficiency: comparison of genotypes at equal shoot P content is critical. Plant Soil 401:79–91. 10.1007/S11104-015-2565-7/TABLES/3

[CR45] Rose TJ, Wissuwa M (2012) Rethinking internal phosphorus utilization efficiency. A new approach is needed to improve PUE in grain crops. Adv Agron 116:183–215

[CR46] Rose TJ, Rose MT, Pariasca-Tanaka J et al (2011) The frustration with utilization: why have improvements in internal phosphorus utilization efficiency in crops remained so elusive? Front Plant Sci 2:1–5. 10.3389/fpls.2011.00073 10.3389/fpls.2011.00073 PMC3355673 22639608

[CR47] Shimamura E, Merckx R, Smolders E (2021) Limited effects of the soluble organic phosphorus fraction on the root phosphorus uptake efficiency of upland rice genotypes grown in acid soil. Soil Sci Plant Nutr 67:120–129. 10.1080/00380768.2020.1864230

[CR48] Shoji S, Nanzyo M, Dahlgren R (1993) Productivity and utilization of volcanic ash soils. In: Shoji S, Nanzyo M, Dahlgren R (eds) Volcanic ash soils genesis, properties and utilization. Elsevier. Amsterdam, pp 209–251

[CR49] Simpson RJ, Oberson A, Culvenor RA et al (2011) Strategies and agronomic interventions to improve the phosphorus-use efficiency of farming systems. Plant Soil 349:89–120. 10.1007/s11104-011-0880-1

[CR50] Takahashi S (2007) Residual effect of phosphorus fertilizer in a low-humic andosol with low extractable phosphorus. Commun Soil Sci Plant Anal 38:1479–1485. 10.1080/00103620701378433

[CR51] Takahashi T, Shoji S (2002) Distribution and classification of volcanic ash soils. Glob Environ Res 6:83–97

[CR52] Tiessen H, Moir JO (2008) Characterization of available P by sequential extraction. In: Carter MR, Gregorich EG (eds) Soil Sampling and Methods of Analysis, 2nd edn. CRC Press, pp 293–306

[CR53] Ulrich AE, Frossard E (2014) On the history of a reoccurring concept: Phosphorus scarcity. Sci Total Environ 490:694–707. 10.1016/j.scitotenv.2014.04.050 24907605 10.1016/j.scitotenv.2014.04.050

[CR54] Vandamme E, Rose T, Saito K et al (2016) Integration of P acquisition efficiency, P utilization efficiency and low grain P concentrations into P-efficient rice genotypes for specific target environments. Nutr Cycl Agroecosystems 104:413–427. 10.1007/s10705-015-9716-3

[CR55] Veneklaas EJ, Lambers H, Bragg J et al (2012) Opportunities for improving phosphorus-use efficiency in crop plants. New Phytol 195:306–320 22691045 10.1111/j.1469-8137.2012.04190.x

[CR56] Wang X, Shen J, Liao H (2010a) Acquisition or utilization, which is more critical for enhancing phosphorus efficiency in modern crops? Plant Sci 179:302–306. 10.1016/J.PLANTSCI.2010.06.007

[CR57] Wang X, Tang C, Guppy CN, Sale PWG (2010b) Cotton, wheat and white lupin differ in phosphorus acquisition from sparingly soluble sources. Environ Exp Bot 69:267–272. 10.1016/j.envexpbot.2010.04.007

[CR58] White PJ, Veneklaas EJ (2012) Nature and nurture: The importance of seed phosphorus content. Plant Soil 357:1–8. 10.1007/s11104-012-1128-4

[CR59] Wissuwa M, Gonzalez D, Watts-Williams SJ (2020) The contribution of plant traits and soil microbes to phosphorus uptake from low-phosphorus soil in upland rice varieties. Plant Soil 448:523–537. 10.1007/s11104-020-04453-z

[CR60] Yoshida S (1981) Fundamentals of rice crop science. Los Ba˜nos Philippines: International Rice Research Institute, p 279

