# Supplementary material for: Fertilizer Addition Modifies Utilization of Different P Sources in Upland Rice on Strongly P-fixing Andosols
Source: J Soil Sci Plant Nutr. 2024 May 7;24(2):3537–49. doi: 10.1007/s42729-024-01774-1 (PMC11636985; doi:10.1007/s42729-024-01774-1)
Supplement: Supplementary file 1 — Supplementary file1 (DOCX 90 KB) [file 42729_2024_1774_MOESM1_ESM.docx]

**Supplementary data**

Suppl. Table S1: Soil properties of the quartz sand and the sandy soil collected from a field near Müncheberg, Germany, used in the sorption experiment.

|  | Clay | Silt | Sand | pH | C_Total_ | N_Total_ | P_Total_ |
| --- | --- | --- | --- | --- | --- | --- | --- |
|  | [%] | [%] | [%] | [CaCl_2_] | [g kg^-1^] | [g kg^-1^] | [mg kg^-1^] |
| Quartz sand | 0 | 0 | 100 | 6.61 | 0.08 | 0.06 | 5 |
| Sandy soil | 4 | 9 | 87 | 4.70 | 4.02 | 0.39 | 439 |

pH analyzed according to DIN ISO 10390; C_Total_ analyzed according to DIN ISO 10694; N_Total_ analyzed according to DIN ISO 13878; P_Total_ according to König (2005).

Suppl. Table S2: Effect of P treatment on biomass and P content 9 days after emergence.

|  |  | Low-P |  | High-P |  |
| --- | --- | --- | --- | --- | --- |
| Plant dry weight | [mg plant^-1^] | 82.0 | a | 78.4 | a |
|  |  | ± 3.5 |  | ± 2.6 |  |
| Shoot dry weight | [mg plant^-1^] | 56.2 | a | 56.1 | a |
|  |  | ± 2.5 |  | ± 2.1 |  |
| Root dry weight | [mg plant^-1^] | 25.8 | a | 22.3 | a |
|  |  | ± 1.6 |  | ± 1.2 |  |
| Root:shoot-ratio |  | 0.46 | a | 0.40 | a |
|  |  | ± 0.026 |  | ± 0.02 |  |
| Plant P content | [µg P plant^-1^] | 104.9 | a | 112.4 | a |
|  |  | ± 3.7 |  | ± 2.9 |  |
| Total P uptake | [µg P plant^-1^] | 40.6 | a | 45.4 | a |
|  |  | ± 4.6 |  | ± 3.0 |  |
| Shoot P content | [µg P shoot^-1^] | 67.4 | b | 74.9 | a |
|  |  | ± 2.4 |  | ± 2.2 |  |
| Root P content | [µg P root^-1^] | 37.5 | a | 37.5 | a |
|  |  | ± 1.9 |  | ± 2.5 |  |
| Shoot P concentration | [mg P g^-1^] | 1.23 | a | 1.35 | a |
|  |  | ± 0.06 |  | ± 0.04 |  |
| Root P concentration | [mg P g^-1^] | 1.55 | a | 1.67 | a |
|  |  | ± 0.05 |  | ± 0.07 |  |
| P utilization efficiency | [mg plant dry weight µg P ^-1^] | 0.782 | a | 0.697 | b |
|  |  | ± 0.024 |  | ± 0.012 |  |

Values are means of the four genotypes ± SE. Different letters indicate significant differences between P-treatments (p < 0.05, Tukey’s HSD, n = 15).

Suppl. Table S3: Plant dry weight and total P uptake of the tested genotypes at the first harvest point 9 DAE.

| **Low-P** |  | AB199 |  | AB67 |  | DJ123 |  | Nerica4 |  |
| --- | --- | --- | --- | --- | --- | --- | --- | --- | --- |
|  |  | (n = 4) |  | (n = 3) |  | (n = 4) |  | (n = 4) |  |
| Shoot dry weight | [mg plant^-1^] | 62.3 | a | 58.3 | ab | 59.8 | ab | 45.0 | b |
|  |  | ± 2.3 |  | ± 4.4 |  | ± 1.8 |  | ± 5.5 |  |
| Root dry weight | [mg plant^-1^] | 27.0 | a | 23.4 | a | 27.3 | a | 24.9 | a |
|  |  | ± 2.7 |  | ± 1.8 |  | ± 1.0 |  | ± 5.6 |  |
| Root:shoot-ratio |  | 0.43 | a | 0.40 | a | 0.46 | a | 0.55 | a |
|  |  | ± 0.04 |  | ± 0.01 |  | ± 0.03 |  | ± 0.08 |  |
| Shoot P concentration | [mg P g^-1^] | 1.16 | a | 1.25 | a | 1.16 | a | 1.34 | a |
|  |  | ± 0.04 |  | ± 0.05 |  | ± 0.06 |  | ± 0.24 |  |
| Root P concentration | [mg P g^-1^] | 1.51 | a | 1.40 | a | 1.54 | a | 1.73 | a |
|  |  | ± 0.10 |  | ± 0.03 |  | ± 0.10 |  | ± 0.06 |  |
| Plant P content | [µg P plant^-1^] | 113.17 | a | 105.27 | a | 111.05 | a | 90.20 | a |
|  |  | ± 9.20 |  | ± 5.68 |  | ± 3.41 |  | ± 4.51 |  |
| Total P uptake | [µg P plant^-1^] | 40.32 | ab | 36.46 | ab | 51.68 | a | 23.15 | b |
|  |  | ± 9.20 |  | ± 5.68 |  | ± 3.41 |  | ± 4.51 |  |
| **High-P** |  | (n = 4) |  | (n = 4) |  | (n = 4) |  | (n = 4) |  |
| Shoot dry weight | [mg plant ^-1^] | 62.5 | a | 52.5 | ab | 61.3 | a | 48.0 | b |
|  |  | ± 3.8 |  | ± 1.4 |  | ± 2.6 |  | ± 3.7 |  |
| Root dry weight | [mg plant ^-1^] | 22.9 | a | 22.0 | a | 21.6 | a | 22.7 | a |
|  |  | ± 1.8 |  | ± 1.1 |  | ± 3.2 |  | ± 3.4 |  |
| Root:shoot-ratio |  | 0.37 | a | 0.42 | a | 0.35 | a | 0.47 | a |
|  |  | ± 0.03 |  | ± 0.03 |  | ± 0.05 |  | ± 0.04 |  |
| Shoot P concentration | [mg P g^-1^] | 1.27 | a | 1.37 | a | 1.28 | a | 1.48 | a |
|  |  | ± 0.04 |  | ± 0.02 |  | ± 0.04 |  | ± 0.14 |  |
| Root P concentration | [mg P g^-1^] | 1.72 | a | 1.83 | a | 1.62 | a | 1.52 | a |
|  |  | ± 0.06 |  | ± 0.17 |  | ± 0.09 |  | ± 0.18 |  |
| Plant P content | [µg P plant^-1^] | 119.18 | a | 112.49 | a | 113.90 | a | 104.11 | a |
|  |  | ± 7.94 |  | ± 5.78 |  | ± 3.73 |  | ± 4.50 |  |
| Total P uptake | [µg P plant^-1^] | 46.34 | a | 43.68 | a | 54.52 | a | 37.07 | a |
|  |  | ± 7.94 |  | ± 5.78 |  | ± 3.73 |  | ± 4.50 |  |

Values are means ± SE. Distinct letters indicate significant differences between genotypes in the low- and high-P treatment, respectively (p < 0.05, Tukey’s HSD).

Suppl. Table S4: Seed dry weight and P content of upland rice genotypes used for the experiments

| Genotype | Seed dry weight |  | Seed-P content |  |
| --- | --- | --- | --- | --- |
|  | [mg] |  | [µg P seed^-1^] |  |
| AB199 | 23.77 | a | 104.06 | a |
|  | ± 0.33 |  | ± 2.17 |  |
| AB67 | 22.41 | ab | 98.30 | ab |
|  | ± 0.95 |  | ± 3.87 |  |
| DJ123 | 23.69 | a | 84.83 | b |
|  | ± 0.73 |  | ± 2.99 |  |
| Nerica4 | 20.31 | b | 95.77 | ab |
|  | ± 0.68 |  | ± 3.15 |  |

Values are means ± SE. Distinct letters indicate significant differences between genotypes (p < 0.05, Tukey’s HSD, n = 3).


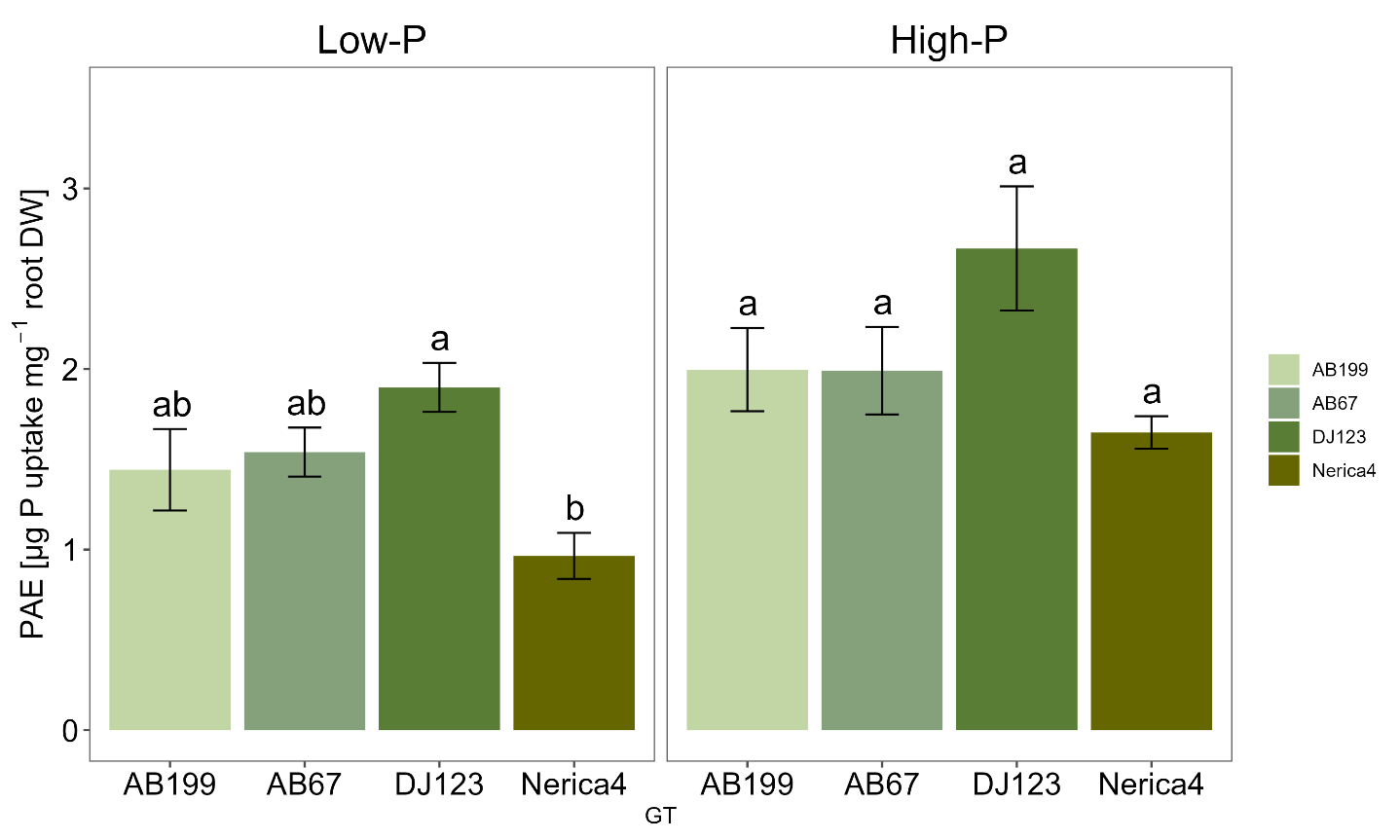


Suppl. Fig. S1: Phosphorous acquisition efficiency (PAE) of the four genotypes AB199, AB67, DJ123 and Nerica4 9 days after emergence under low- and high-P conditions. Variation is given as SE, n = 4 and 3. Distinct letters indicate significant differences between genotypes (p < 0.05, Tukey’s HSD) in the low- and high-P treatment, respectively.

Suppl. Table S5: Effect of P treatment on biomass and P content 34 days after emergence.

|  |  | Low-P |  | High-P |  |
| --- | --- | --- | --- | --- | --- |
| Plant P content | [mg P plant^-1^] | 0.224 | b | 1.65 | a |
|  |  | ± 0.012 |  | ± 0.12 |  |
| Total P uptake | [mg total P uptake plant^-1^] | 0.143 | b | 1.57 | a |
|  |  | ± 0.013 |  | ± 0.12 |  |
| Shoot P concentration | [mg P g^-1^] | 0.58 | b | 1.67 | a |
|  |  | ± 0.02 |  | ± 0.04 |  |
| P fertilizer use efficiency (PFUE) | [%] | 0.35 | b | 2.23 | a |
|  |  | ± 0.04 |  | ± 0.16 |  |
| P derived from seed (PdfSeed) | [%] | 38.1 | a | 5.4 | b |
|  |  | ± 2.3 |  | ± 0.4 |  |
| P derived from fertilizer (PdfF) | [%] | 3.8 | b | 66.9 | a |
|  |  | ± 0.3 |  | ± 0.6 |  |
| P derived from soil (PdfS) | [%] | 58.2 | a | 27.8 | b |
|  |  | ± 2.2 |  | ± 0.8 |  |
| Quantity of PdfSeed | [mg seed-P uptake plant^-1^] | 0.0814 | a | 0.0814 | a |
|  |  | ± 0.0015 |  | ± 0.0015 |  |
| Quantity of PdfF | [mg fertilizer-P uptake plant^-1^] | 0.009 | b | 1.102 | a |
|  |  | ± 0.001 |  | ± 0.080 |  |
| Quantity of PdfS | [mg native soil-P uptake plant^-1^] | 0.134 | b | 0.468 | a |
|  |  | ± 0.013 |  | ± 0.046 |  |
| Fertilizer-PAE | [mg fertilizer-P uptake g^-1^ root DW] | 0.053 | b | 3.003 | a |
|  |  | ± 0.005 |  | ± 0.088 |  |
| Soil-PAE | [mg native soil-P uptake g^-1^ root DW] | 0.803 | b | 1.246 | a |
|  |  | ± 0.049 |  | ± 0.047 |  |

Values are means of the four genotypes tested ± SE. Distinct letters indicate significant differences between P-treatments (p < 0.05, Tukey’s HSD, n = 16).
